# Dr. Alder, on the Proximate Causes of Diseases; in Continuation of His Letters on Quarantines, Contagion, and Fevers

**Published:** 1808-04

**Authors:** 


*Dr.* ALDER, *on the proximate Causes of Diseases; in Continuation of his Letters on Quarantines, Contagion, and Fevers.* pp. 198—204.

IN proposing doctrines materially new, we may be supposed to attack, *in some degree,* such as are established; I shall therefore preface and apologize for the very great *innovations* which this letter is to contain, by a quotation which has been thought so pure and generally applicable, by a man who has met with universal esteem, that it was by him addressed to *youth* of all descriptions, "Do not think learning, in general, is arrived at its perfection, or that the knowledge of any *particular* subject, in any science, cannot be improved; merely because it has lain five hundred or a thousand years without improvement." By considering only how many great men oppose each other, and occasionally themselves, particularly on the present subject, we shall find, *Authority* is not entitled to the smallest degree of confidence and unscrutinizing veneration, any more than *Antiquity,*—Demonstration being, *incomparably,* superior to both.

But to proceed.—It is not with an intention to censure gentlemen for believing the theory of oxygenation, that in my last I disclaim it; there are some pretended facts whch which as naturally lead to it as Dr. Crawford's experiments do to that of animal heat; though it, as well as Dr. Crawford's theory, will not explain the phænomena of vitality, and particularly such as occur *in diseases*. These facts are placed, by the learned and industrious compiler of the Medical Extracts, in pages 71 and 72, in the third volume of that work;—there are two experiments (by whom made I cannot learn) in which vital air in great quantity, in a free form, was disengaged spontaneously from arterial blood! and these, if true, certainly would prove that the arterial blood actually is oxygenated; but they stand not only unsupported by any other experiments, but generally contradicted.

It appears too, warm blood becomes red on the admission of oxygen, even through a bladder; but this *of itself* proves little, as the blood often becomes red when no oxygen is added to it; and, on the other hand, often black when none is subtracted.

It seems also that *part* of the oxygen inspired actually enters into the blood vessels. But this is no more calculated than the last to demonstrate the now fashionable doctrines of the oxygenation of the blood; as it must either circulate in a free state in the vessels, or combine; but if it circulates in a free state, it would very soon inflame the vessels; and if in a combined state, it would lose all the properties of vital air.

But the strongest arguments against the doctrine of the oxygenation of the system, are, 1st. This doctrine will by no means explain the phænomena of disease. And, 2dly, The system is oxygenated and de-oxygenated in innumerable cases (as from various foods, drinks, &c.) for a length of time, to a great extent, without any such effects as should result, were the doctrines of oxygenation true; and very often indeed with such as are opposite to them. Thus people may live on oxygenous vegetables, or vinegar, without any increase of heat or action, but with a great *decrease* of both; and they may live on azotic or hydrogenous food and drink, without any *decrease* of heat and action; nay, with a very considerable increase of them.

It is true, the oxygenation advocates endeavour to explain some of these objections away, by alleging there is a great increase of appetite for oxygen, when the system is de-oxygenated, and a greater consumption of it at the lungs; and that, when the system is oxygenated by vegetable food or acids, there is a less demand for oxygen at the lungs, and a decreased consumption of it there. But these these circumstances (however true) are by no means capable of removing such weighty objections; as, according to their theory, the system must *(notwithstanding them)* be greatly de-oxygenated, when the heat and action in it are increased, and considerably oxygenated when these are diminished.

But the first of these two latter objections, viz. that the oxygenatic doctrine will not explain the phænomenon of diseases, is perhaps as strong against it as any other, and is equally as much to be noticed. We find, in endless examples of disease, especially in fevers, a prodigious increase of heat and action, after the lungs have been oppressed in a cold fit, that so far from having taken up more oxygen into the system, they could scarcely have taken in any; and, indeed, sometimes the atmospheric air itself in such a case is almost totally excluded from them. I have heard attempts to explain the increase of heat and action which takes place after the cold fit of fever, by some of the oxygenationists, on the principle too that the system in the cold fit had a greater *appetite* for air; but such attempts are, to my judgment, perfectly ridiculous; because, if the appetite be ever so much increased, it cannot be more indulged, as the quantity of air *admitted into the lungs* in the cold fit, really is exceedingly small. It is true, the same phænomena militate against Dr. Crawford's Theory of Animal Heat; and this may be said to be established on the basis of clear induction from facts. But to this I shall answer, that Dr. Crawford's experiments have not been found accurate by others; and that this circumstance alone of their being so broadly and clearly contradicted by morbid phænomena, is sufficient evidence with me that they are fallacious; and I have heard it said the Doctor himself found them so, but suspect there must on this head have been some mistake.

It will be seen therefore, I hope, in the first instance, that so far from building any thing upon ingenious theories formed by other people, I do not receive those theories. Indeed it is a pleasing subject for reflection to myself, that I have not admitted one theory which is not fully proved and universally acknowledged to be true.

But theories were not wanted; for, in forming the following inferences, I had only to look at some plain and well-known facts; and that any well educated medical man could avoid forming them, would appear to me almost miraculous, *(they are so very evident)* did I not recollect the very slow progress I have made in advancing to them myself, myself, notwithstanding that I was fortunate enough to get the clue, at a very early period of life, which leads to them. This is owing to the false doctrines and false facts which we have been overloaded with from infancy, and by the stupor which we are thrown into by these, and the veneration for authority.

We know the blood will not pass through the lungs long together, except a due quantity of oxygen be applied to the vessels contained in it. And this simple circumstance, duly reflected on, will lead us to the proximate causes of most diseases; for when the blood cannot pass freely through the lungs, it cannot come freely from the head, from the liver and intestines, that is, from the veins, and it cannot pass freely into the arteries. HENCE THE PRINCIPAL PHÆNOMENA WHICH TAKE PLACE IN MOST DISEASES, AND ESPECIALLY IN FEVERS.

This, Gentlemen, appears reducing the theory of medicine into an exceedingly short compass, and may on that account subject me to a little ridicule;—but is this theory of the proximate causes of diseases more simple than that of gravitation? Or, than that in our own profession, on which it is principally formed; I mean, that of the circulation? Is it too, more simple than the new chemical doctrines of inanimate oxygenation?—I shall not, however, attempt to recommend it at present, or to maintain it, or enlarge upon it. But, after calling upon gentlemen to recollect here, what I have said in my former letters about epidemic, endemic, and sporadic *bad air,* applies here, to shew in what innumerable instances of epidemic, endemic, and sporadic diseases, oxygen is not applied to the lungs in its usual and due quantity; I shall, for the present, leave this theory of the proximate causes of diseases, and particularly of fevers, to its natural fate.

*March* 9, 1808.

( To be continued. )

